# Early Indicators of Fatal Leptospirosis during the 2010 Epidemic in Puerto Rico

**DOI:** 10.1371/journal.pntd.0004482

**Published:** 2016-02-25

**Authors:** Tyler M. Sharp, Brenda Rivera García, Janice Pérez-Padilla, Renee L. Galloway, Marta Guerra, Kyle R. Ryff, Dana Haberling, Sharada Ramakrishnan, Sean Shadomy, Dianna Blau, Kay M. Tomashek, William A. Bower

**Affiliations:** 1 Epidemic Intelligence Service, Centers for Disease Control and Prevention, Atlanta, Georgia, United States of America; 2 Dengue Branch, Division of Vector-Borne Diseases, Centers for Disease Control and Prevention, San Juan, Puerto Rico; 3 Puerto Rico Department of Health, San Juan, Puerto Rico; 4 Bacterial Special Pathogens Branch, Division of High Consequence Pathogens, Centers for Disease Control and Prevention, Atlanta, Georgia, United States of America; 5 Infectious Diseases Pathology Branch, Division of High Consequence Pathogens and Pathology, Centers for Disease Control and Prevention, Atlanta, Georgia, United States of America; University of California San Diego School of Medicine, UNITED STATES

## Abstract

**Background:**

Leptospirosis is a potentially fatal bacterial zoonosis that is endemic throughout the tropics and may be misdiagnosed as dengue. Delayed hospital admission of leptospirosis patients is associated with increased mortality.

**Methodology/Principal Findings:**

During a concurrent dengue/leptospirosis epidemic in Puerto Rico in 2010, suspected dengue patients that tested dengue-negative were tested for leptospirosis. Fatal and non-fatal hospitalized leptospirosis patients were matched 1:1–3 by age. Records from all medical visits were evaluated for factors associated with fatal outcome. Among 175 leptospirosis patients identified (4.7 per 100,000 residents), 26 (15%) were fatal. Most patients were older males and had illness onset during the rainy season. Fatal case patients first sought medical care earlier than non-fatal control patients (2.5 vs. 5 days post-illness onset [DPO], p < 0.01), but less frequently first sought care at a hospital (52.4% vs. 92.2%, p < 0.01). Although fatal cases were more often diagnosed with leptospirosis at first medical visit (43.9% vs. 9.6%, p = 0.01), they were admitted to the hospital no earlier than non-fatal controls (4.5 vs. 6 DPO, p = 0.31). Cases less often developed fever (p = 0.03), but more often developed jaundice, edema, leg pain, hemoptysis, and had a seizure (p ≤ 0.03). Multivariable analysis of laboratory values from first medical visit associated with fatal outcome included increased white blood cell (WBC) count with increased creatinine (p = 0.001), and decreased bicarbonate with either increased WBC count, increased creatinine, or decreased platelet count (p < 0.001).

**Conclusions/Significance:**

Patients with fatal leptospirosis sought care earlier, but were not admitted for care any earlier than non-fatal patients. Combinations of routine laboratory values predictive of fatal outcome should be considered in admission decision-making for patients with suspected leptospirosis.

## Author Summary

Leptospirosis is a common tropical illness that results from exposure to the urine of animals infected with *Leptospira* bacteria. Because leptospirosis shares signs and symptoms with other common tropical illnesses such as dengue, identification of patients with leptospirosis can be challenging. Early identification of patients with leptospirosis is necessary to initiate antibiotic therapy and in some cases provide in-hospital management. During an epidemic of leptospirosis in Puerto Rico that occurred during a concomitant dengue epidemic, we identified leptospirosis patients by screening specimens from suspected dengue patients. Of 175 leptospirosis patients identified, 26 (15%) died. After comparing leptospirosis patients that died to patients of a similar age that were hospitalized but survived, we observed that fatal cases were more often sent home after their first medical visit. We next identified several routinely available laboratory values from patients’ first medical visit that were associated with patients that died. Clinicians can use such laboratory values to diagnose and hospitalize leptospirosis patients at increased risk for fatal outcome.

## Introduction

Leptospirosis is an emerging zoonosis caused by infection with bacterial spirochetes of the genus *Leptospira*, and is endemic throughout the tropics where >1 million cases and ~60,000 deaths occur annually [1, 2]. Human infection typically occurs through direct or indirect contact with the urine of infected animals [1]. Leptospirosis is typically a mild acute febrile illness (AFI); however, ~10% of patients progress to severe leptospirosis with acute kidney failure, jaundice, and/or pulmonary hemorrhage [1, 3]. The case-fatality rate for patients with severe leptospirosis ranges from 5–20% [4–6].

Due to similar clinical presentations, leptospirosis may be misdiagnosed as dengue [7–9]. Delayed or misdiagnosis of leptospirosis patients has been associated with increased mortality, potentially due to delayed administration of antibiotics [10–15]. Therefore, identification of early clinical markers of patients at risk for severe disease to thereby enable earlier patient admission may result in improved outcome. Severe thrombocytopenia, increased serum creatinine or BUN, hemoptysis, dyspnea, and jaundice have been associated with severe or fatal outcome in leptospirosis patients [5, 12, 14–18]; however, few studies have captured data from patients’ entire clinical course to identify demographic characteristics, clinical findings, or missed opportunities in clinical management associated with poor outcome [12, 14]. Consequently, early clinical indicators of patients that have or will develop severe disease have not been well elucidated.

During 1990–2014, a total of 729 leptospirosis cases were reported to Puerto Rico Department of Health (PRDH), of which 78 (10.7%) were fatal (S1 Fig). Such surveillance enabled documentation of leptospirosis epidemics in 2006, 2007, and 2010. However, because of underreporting of leptospirosis [19], which is attributable in part to misdiagnosis as dengue [20–22], it is unclear if these data represent the true epidemiologic trends of leptospirosis. Factors associated with severe or fatal outcome in leptospirosis patients have not previously been investigated in Puerto Rico.

To better understand the epidemiology of leptospirosis during the 2010 dengue epidemic in Puerto Rico [23], we conducted enhanced surveillance by performing leptospirosis diagnostic testing on specimens from suspected dengue patients. We also reviewed medical records from all health care visits of identified leptospirosis patients to identify demographic characteristics, clinical signs and symptoms, laboratory values, and clinical practices associated with fatal outcome.

## Methods

### Ethics statement

This study was approved by the Institutional Review Board at the Centers for Disease Control and Prevention (CDC) (protocol # 6285).

### Data sources and diagnostic testing

Leptospirosis cases in Puerto Rico in 2010 were identified from four sources. First, suspected dengue cases reported via the Passive Dengue Surveillance System (PDSS) [24] that had no evidence of dengue virus (DENV) infection by rRT-PCR or anti-DENV IgM ELISA [23] (N = 2,519) were eligible to be tested for evidence of *Leptospira spp*. infection by microscopic agglutination test (MAT) [25] and polymerase chain reaction (PCR) with primers specific for *Leptospira* spp. *LipL32* [26]. Specimens selected for leptospirosis testing (n = 1,133) came from cases for which either: a) paired acute and convalescent specimens were available (n = 654); or b) only a convalescent specimen was available and the case had reported fever, body pain or headache, and jaundice, hemorrhage, or pleural effusion (n = 479). Second, fatal leptospirosis cases were identified via the Enhanced Fatal AFI Surveillance System (EFASS) in which: a) serum or tissue specimens collected during autopsy were tested by MAT, PCR, or immunohistochemistry (IHC) [27]; and b) death certificates were reviewed for use of “leptospirosis” or “Weil’s disease”. Third, all leptospirosis cases reported to PRDH along with a positive diagnostic test result via the Notifiable Diseases Surveillance System (NDSS) were included. Last, two commercial laboratories were queried for leptospirosis cases that tested positive by IgM dot blot. Cases identified through more than one data source with matching names and dates of birth were considered a single case.

### Definitions

A *laboratory-positive leptospirosis patient* was defined as a person that had evidence of infection with *Leptospira spp*. by detection of: i) antigen in a tissue specimen by IHC; ii) nucleic acid in a serum or tissue specimen by PCR; iii) ≥4-fold rise in MAT titer in paired serum specimens; iv) MAT titer ≥800 in a single serum specimen; v) anti-*Leptospira* IgM antibody at a private laboratory; or vi) MAT titer ≥100 but <800 in a single serum specimen. A *confirmed leptospirosis patient* was defined by any of criteria i–v; a *probable leptospirosis patient* was defined by criteria vi. A *suspected fatal leptospirosis patient* was a person who died in Puerto Rico in 2010, had the word “leptospirosis” written on the death certificate, and had either: a) no leptospirosis diagnostic testing performed; or b) negative diagnostic testing performed at a commercial laboratory on a specimen collected within five days of illness onset.

### Case-control study

Each fatal, laboratory-positive leptospirosis patient (i.e., cases) was matched by age within five years with up to three non-fatal, hospitalized, laboratory-positive leptospirosis patients (i.e., controls). All available medical records–including private office, out-patient clinic, emergency department, and inpatient hospitalizations–during the episode of illness were reviewed. Controls that left the hospital against medical advice or had incomplete medical records were replaced.

### Data analysis

The frequencies of clinical, demographic and laboratory data were calculated by performing descriptive analyses of all leptospirosis patients identified in 2010 and compared using Student’s t-test or Chi squared test. Rates of leptospirosis by age group and municipality of residence were calculated using data from the 2010 United States Census [28]. Statistical differences and modeling of matched case-control data were performed using exact conditional logistic regression. Due to a limited number of matched pairs, several combinations of clinical lab results were considered for independent predictors of fatal outcome. Normal limits of laboratory values were defined by accepted standards [29].

All data analyses were conducted using SAS version 9.3 (SAS Institute Inc., Cary, NC), graphs were produced in SAS and Microsoft Excel (Microsoft Corp., Redmond, WA), and maps were created using ArcView (ESRI, Redlands, CA). Specimens were not anonymized prior to diagnostic testing to enable reporting of results to requesting physicians. Data included in the case-control study were anonymized prior to analysis.

## Results

### Identification of leptospirosis patients

Among 1,133 suspected but laboratory-negative dengue cases that were selected for leptospirosis diagnostic testing, 105 (9.3%) were laboratory-positive (S1 Table). Among 802 specimens from patients tested for leptospirosis at a private laboratory, 56 (7.0%) were positive. A total of 57 non-fatal leptospirosis patients were reported via NDSS in 2010, and laboratory diagnostic evidence was provided for 15 (26%). After consolidating individual patients identified by multiple systems, a total of 149 non-fatal, laboratory-positive leptospirosis patients were identified in Puerto Rico in 2010 (4.0 non-fatal patients per 100,000 residents), of which 91 (61%) were confirmed and 58 (39%) were probable leptospirosis patients. Dengue was ruled out for 134 (90%) non-fatal leptospirosis patients by rRT-PCR and/or IgM ELISA [23]; one apparent co-infection was identified in which DENV-1 was detected by RT-PCR and anti-*Leptospira spp*. IgM antibody was detected at a private laboratory.

A total of 26 fatal leptospirosis patients were identified (0.7 fatal patients per 100,000 residents), of which 21 were confirmed and five were suspected leptospirosis patients; only two (7.7%) had been reported to PRDH. Fifteen fatal, laboratory-positive leptospirosis patients had available kidney and liver tissue specimens, and *Leptospira* antigen was detected by IHC in all 15. Dengue was ruled out in 18 (86%) of the fatal, laboratory-positive leptospirosis patients and in two (40%) of the fatal, suspected leptospirosis patients. Two patients with fatal DENV/*Leptospira spp*. co-infection were identified [30]. Among all 26 fatal leptospirosis patients, the most common reported causes of death included respiratory, cardiac, or renal failure, and septic shock (S2 Table).

MAT-positive specimens (n = 130) from laboratory-positive leptospirosis patients showed strongest reactivity to serogroups including Icterohaemorrhagiae (57%), Australis (11%), Mini (5%), Bataviae (4%), Canicola (4%), Cynopteri (2%), Pyrogenes (2%), Pomona (1%), Djasiman (1%), and Autumnalis (1%); 12% had strongest reactivity against more than one serogroup. Of four PCR-positive serum specimens from one fatal and three non-fatal patients, multi-locus sequence typing [31] identified six of seven alleles suggestive of *L*. *interrogans* serovar Icterohaemorrhagiae/Copenhageni in the specimen from the fatal patient; MLST was not successful for the other specimens.

### Demographics and epidemiology

**Fig 1:**
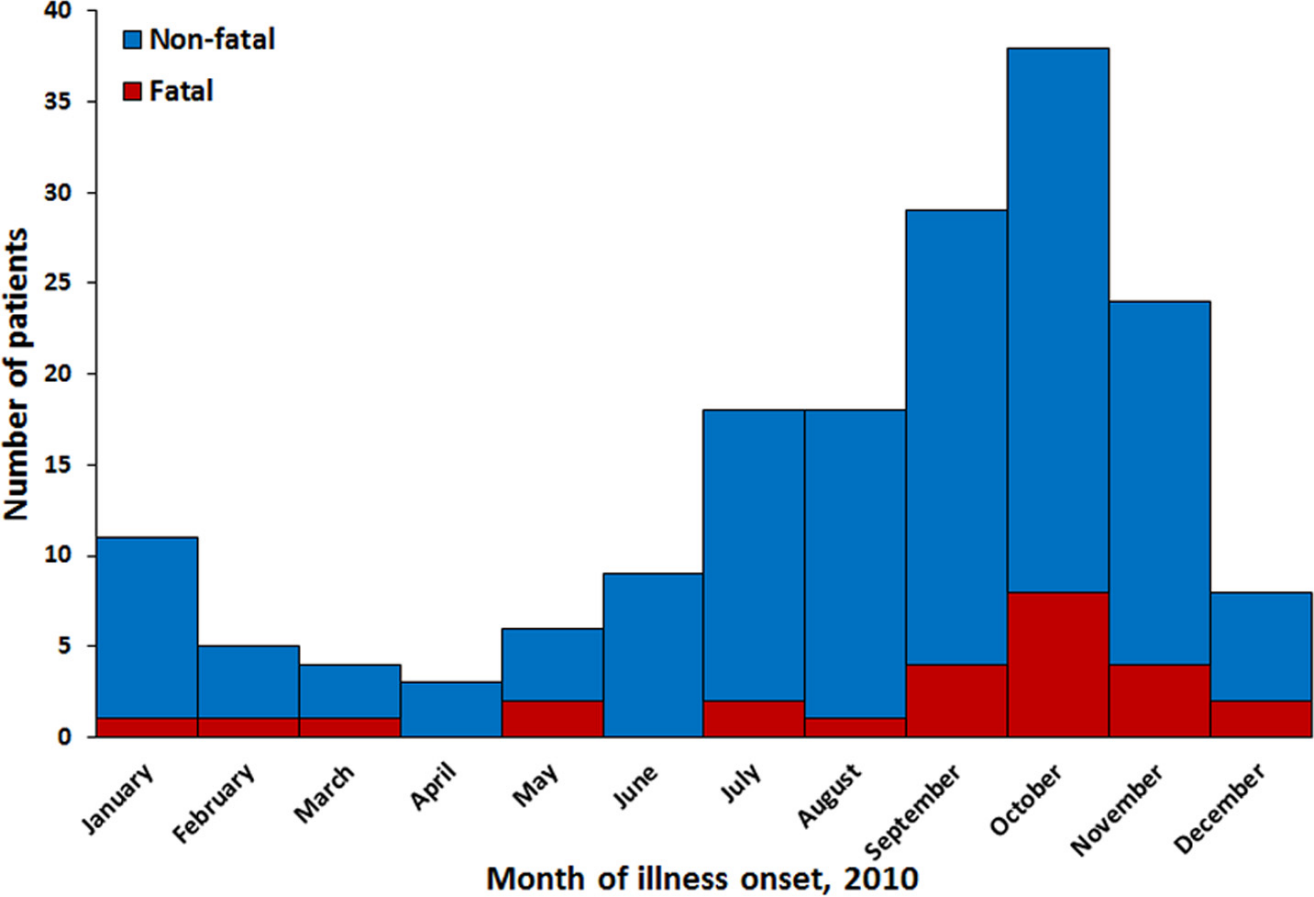
Date of illness onset of fatal (n = 26) and non-fatal (n = 147) leptospirosis patients identified in Puerto Rico, 2010*. *If date of illness onset was unavailable, date of first specimen collection was used instead. Two non-fatal patients had no available date of illness onset or date of specimen collection.

**Fig 2:**
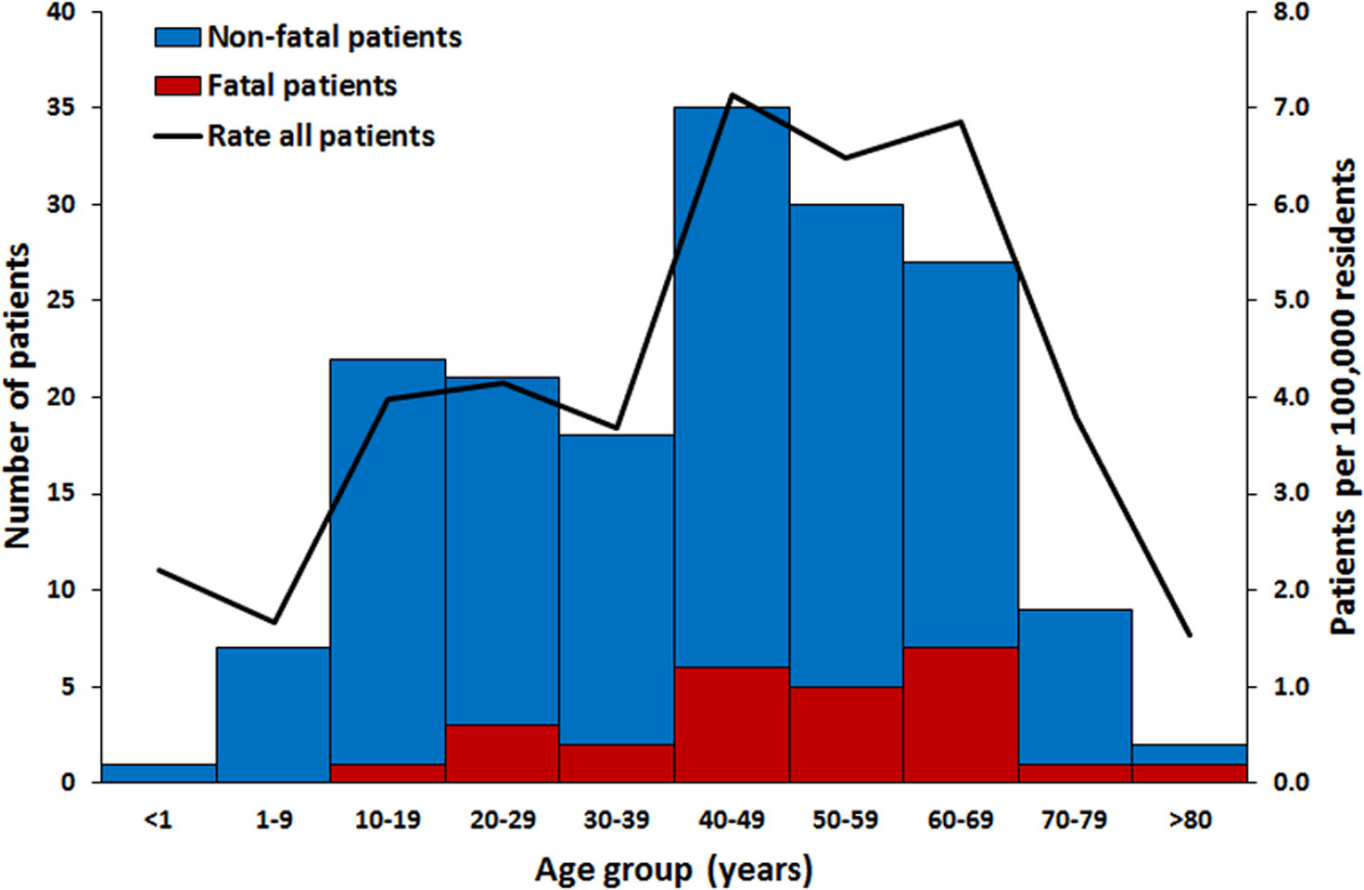
Age group of fatal (n = 26) and non-fatal (n = 146*) leptospirosis patients in Puerto Rico, 2010. Red bars represent fatal laboratory-positive (n = 21) and suspected (n = 5) leptospirosis patients; blue bars represent non-fatal probable (n = 58) and confirmed (n = 88) leptospirosis patients. *age was unavailable for 1 non-fatal patient.

Leptospirosis patients had illness onset in all months of the year (Fig 1). Peak incidence of identified fatal and non-fatal leptospirosis patients occurred in October, in association with the rainy season. Most (79%) fatal and non-fatal laboratory-positive leptospirosis patients were male. Leptospirosis patients were identified in all age groups (Fig 2). Incidence was highest in individuals aged 40–69 years and lowest in individuals aged >80 years. Fatal patients were significantly older than non-fatal patients (mean of 50 vs. 41 years; p = 0.02). Confirmed and probable non-fatal leptospirosis patients were not significantly different by age (p = 0.34) or month of illness onset (p = 0.35); however, more confirmed than probable non-fatal patients were male (85% vs. 68%; p = 0.02). Most non-fatal (59%) and fatal (92%) leptospirosis cases were reported to have been hospitalized. Mortality by age group was highest in those aged 60–69 years (1.8 per 100,000 residents).

**Fig 3:**
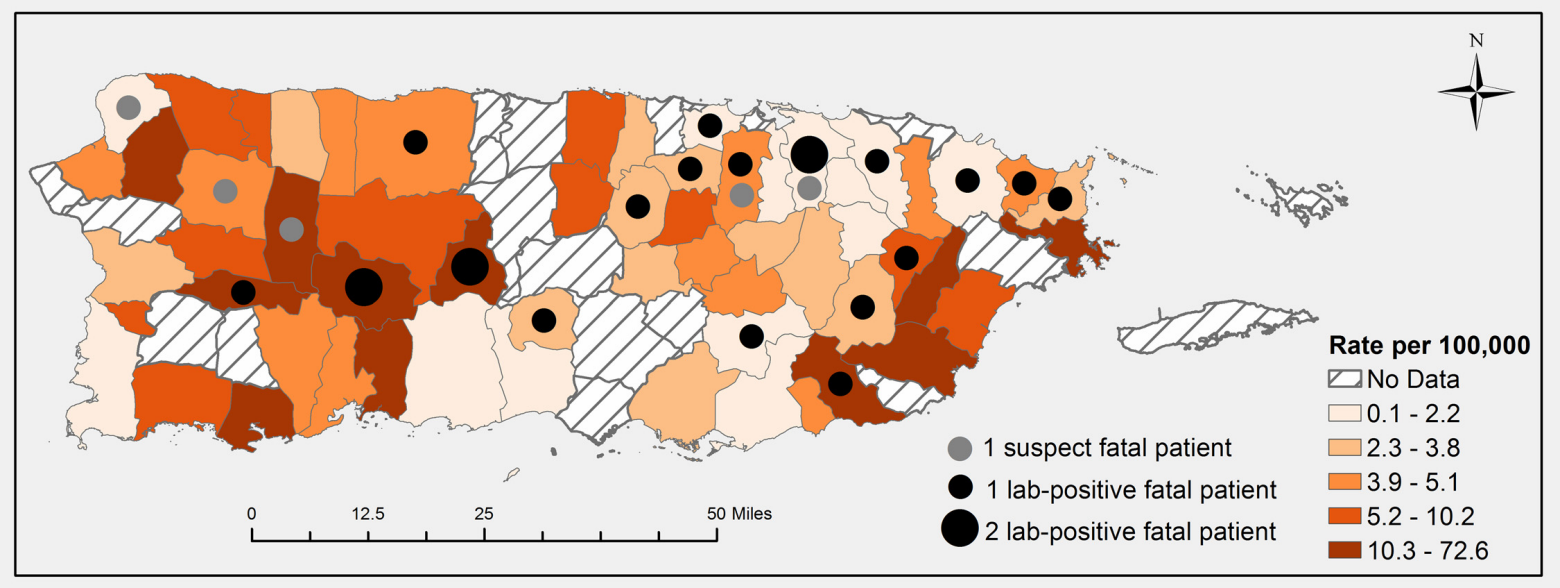
Rate of leptospirosis patients (N = 155*) and number of fatal patients (n = 26) by municipality of residence, Puerto Rico, 2010. Rates were calculated by dividing case numbers by municipality-specific populations, and grouped by quintile. *municipality of residence was unavailable for 18 non-fatal patients

Fatal and non-fatal leptospirosis cases resided in both urban and rural municipalities across Puerto Rico (Fig 3). In the 59 (76%) municipalities for which cases were detected, incidence was highest in Patillas in the rainy southeast–where enhanced dengue surveillance was conducted at a community health center in 2010 [23]–and in the mountainous, agricultural center of the island. Incidence was lowest in Cabo Rojo in the arid southwest.

### Risk factors for fatal outcome

A case-control study was conducted in which data from medical records were compared between 21 laboratory-positive fatal cases and 52 age-matched, laboratory-positive, hospitalized but non-fatal leptospirosis controls. Cases and controls did not differ significantly by sex, occupation, or animal or environmental exposure history, nor by reported co-morbidities or chronic medical conditions (S3 Table).

Fatal cases first sought medical care sooner after illness onset than non-fatal controls, and more often sought care at a private or out-patient clinic (Table 1). Although controls first sought medical care at a hospital more frequently than cases, cases were more often admitted or referred for admission at the first visit. Cases and controls did not differ by day post-illness onset (DPO) of hospitalization or duration of hospital stay. Cases were more often admitted to the intensive care unit, intubated, and received hemodialysis (p ≤ 0.02). Blood products were administered to more than half of cases and controls.

**Table 1:** Characteristics of clinical management of fatal and non-fatal leptospirosis patients, Puerto Rico, 2010.

	Fatal cases	Non-fatal controls	
	N = 21	N = 52	
	n (%)	n (%)	P-value
**Medications taken before first seeking care**			
Acetaminophen	9 (42.9)	16 (30.8)	0.42
Antibiotic	0 (0.0)	5 (9.6)	0.33
Other*	7 (33.3)	11 (21.2)	0.38
**First medical visit**			
**DPO, median (range)**	2.5 (0–10)	5 (2–14)	**<0.01**
**Facility type**			
Private Clinic	2 (9.5)	2 (3.9)	0.62
Out-patient clinic	8 (38.1)	2 (3.9)	Reference
Hospital	11 (52.4)	47 (92.2)	**<0.01**
**Outcome**			
Sent home	8 (38.1)	44 (84.6)	**<0.01**
Admitted or referred for admission	13 (61.9)	8 (15.4)	Reference
**Hospitalization**			
DPO admitted	4.5 (2–10)	6 (2–14)	0.31
Duration of hospitalization in days, median (range)	4 (1–37)	8 (0–150)	0.68
Admitted to intensive care unit	16 (76.2)	21 (40.4)	**0.01**
Intubated	19 (90.5)	7 (13.5)	**<0.01**
Hemodialysis initiated	7 (33.3)	4 (7.7)	**0.02**
Received a blood product^†^	13 (61.9)	28 (53.8)	0.57
**Diagnosis**			
“Leptospirosis” in differential at first medical visit	9 (42.9)	5 (9.6)	**0.01**
“Dengue” or “viral syndrome” in differential at first medical visit	15 (71.4)	31 (59.6)	0.25
“Leptospirosis” in discharge diagnosis at first medical visit	4 (19.0)	14 (26.9)	0.76
“Dengue” or “viral syndrome” in discharge diagnosis at first medical visit	7 (33.3)	26 (50.0)	0.18
“Leptospirosis” mentioned in any medical record	18 (85.7)	31 (59.6)	**0.03**
DPO first mentioned, median (range)	5 (2–10)	7 (2–26)	0.09
DPH first mentioned, median (range)	0.5 (0–20)	1 (0–15)	0.30
“Dengue” mentioned in any medical record	15 (71.4)	52 (100)	**<0.01**
DPO first mentioned, median (range)	3 (1–11)	5 (2–14)	0.23
DPH first mentioned, median (range)	0 (0–4)	0 (0–2)	0.08
**Medications**			
Penicillin-derivative antibiotic	19 (90.5)	38 (73.1)	0.62
DPO given, median (range)	4.5 (3–10)	6.0 (3–13)	0.23
DPH given, median (range)	1 (0–3)	1.0 (0–6)	0.73
Corticosteroid	9 (42.9)	33 (63.5)	0.82
DPO given, median (range)	4 (1–7)	6.0 (2–14)	0.20
DPH given, median (range)	0 (0–2)	0 (0–4)	0.58

*Other = aspirin, non-steroidal anti-inflammatory drug, statin, anti-depressant, anti-histamine, histamine H2R antagonist, nitrate, sulfonylurea

^†^Cases: 8 (38%) received platelets, 5 (24%) received packed red blood cells, and 4 (19%) received fresh frozen plasma; Controls: 18 (35%) received platelets, 15 (29%) received packed red blood cells, 2 (4%) received fresh frozen plasma, and 1 (2%) received plasma.

Abbreviations: DPO = day post-illness onset; DPH = day post-illness onset of hospitalization; N = Normal

Cases more often had leptospirosis included in the differential diagnosis at first medical visit (p = 0.01), whereas controls more often had “dengue” ever mentioned in any medical record (p < 0.01). The timing with which “leptospirosis” and “dengue” were mentioned post-illness onset and post-hospitalization did not differ between cases and controls. Antibiotics were administered to >70% of cases and controls. Corticosteroids were administered to roughly half of cases and controls, most frequently on the day of admission. The frequency, clinical setting (e.g., out-patient clinic vs. hospital), and timing of administration of both antibiotics and corticosteroids did not significantly differ between cases and controls.

Cases presented to first medical visit with either fever or cough less often than controls (Table 2). Similarly, cases less often developed fever throughout hospitalization. Most cases developed jaundice, edema, leg pain, hemoptysis, and altered mental status, while fewer than half of controls had these findings. Developing cyanosis and having a seizure were also associated with fatal outcome.

**Table 2:** Signs and symptoms of fatal (n = 21) and non-fatal (n = 52) leptospirosis patients at first presentation and during hospitalization, Puerto Rico, 2010.

Signs or symptom	At first presentation			During hospitalization		
	Fatal	Non-fatal	P-value	Fatal	Non-fatal	P-value
	(N = 21)	(N = 52)		(N = 21)	(N = 52)	
	n (%)	n (%)		n (%)	n (%)	
Fever*	10 (47.6)	31 (59.6)	**0.01**	15 (71.4)	49 (94.2)	**0.03**
Weakness/lethargy	9 (42.9)	27 (51.9)	0.58	19 (90.5)	46 (88.5)	0.29
Headache	9 (42.9)	26 (50.0)	0.13	14 (66.7)	41 (78.8)	1.00
Eye pain	2 (9.5)	4 (7.7)	0.58	5 (23.8)	9 (17.3)	0.34
Conjunctival suffusion	0 (0.0)	8 (15.4)	0.07	3 (14.3)	12 (23.1)	0.67
Conjunctival hemorrhage	0 (0.0)	2 (3.8)	1.00	2 (9.5)	4 (9.6)	0.58
Icteric sclera	6 (28.6)	10 (19.2)	1.00	14 (66.7)	21 (40.4)	0.37
Jaundice	6 (28.6)	11 (21.2)	0.65	17 (81.0)	22 (42.3)	**0.02**
Cyanosis	2 (9.5)	0 (0.0)	0.08	6 (28.6)	1 (1.9)	**<0.01**
Petechia	1 (4.8)	9 (17.3)	0.65	6 (28.6)	15 (28.8)	0.76
Purpura	3 (14.3)	3 (5.8)	0.17	5 (23.8)	8 (15.4)	1.00
Rash	1 (4.8)	9 (17.3)	1.00	3 (14.3)	16 (30.8)	0.14
Dehydration	8 (38.1)	22 (42.3)	0.06	16 (76.2)	41 (78.8)	1.00
Edema	5 (23.8)	10 (19.2)	0.70	16 (76.2)	16 (30.8)	**<0.01**
Effusion	3 (14.3)	10 (19.2)	0.61	7 (33.3)	18 (34.6)	1.00
Ascites	2 (9.5)	4 (7.7)	1.00	4 (19.0)	5 (9.6)	0.31
Cardiac effusion	1 (4.8)	1 (1.9)	0.33	1 (4.8)	1 (1.9)	0.33
Anorexia	6 (28.6)	19 (36.5)	0.58	13 (61.9)	32 (61.5)	0.48
Vomiting	10 (47.6)	18 (34.6)	1.00	16 (76.2)	30 (57.7)	0.14
Diarrhea	10 (47.6)	14 (26.9)	0.53	15 (71.4)	29 (55.8)	0.13
Hepatomegaly	2 (9.5)	6 (11.5)	1.00	4 (19.0)	10 (19.2)	1.00
Splenomegaly	1 (4.8)	5 (9.6)	1.00	1 (4.8)	8 (15.4)	0.66
Muscle pain	15 (71.4)	32 (61.5)	1.00	19 (90.5)	49 (94.2)	1.00
Leg pain	7 (33.3)	11 (21.2)	0.25	15 (71.4)	20 (38.5)	**0.01**
Dyspnea	6 (28.6)	14 (26.9)	0.66	18 (85.7)	27 (51.9)	0.07
Cough	2 (9.5)	15 (28.8)	**0.01**	8 (38.1)	30 (57.7)	0.17
Altered mental status	6 (28.6)	7 (13.5)	0.45	18 (85.7)	12 (23.1)	**<0.01**
Meningitis	0 (0.0)	0 (0.0)	—^†^	0 (0.0)	1 (1.9)	—^†^
Encephalitis	2 (9.5)	0 (0.0)	0.50	2 (9.5)	1 (1.9)	0.17
Seizure	2 (9.5)	1 (1.9)	1.00	5 (23.8)	1 (1.9)	**0.03**
Epistaxis	1 (4.8)	1 (1.9)	1.00	5 (23.8)	3 (5.8)	0.10
Hematemesis	1 (4.8)	2 (3.8)	1.00	5 (23.8)	7 (13.5)	0.67
Hemoptysis	2 (9.5)	4 (7.7)	0.50	11 (52.4)	8 (15.4)	**0.03**
Hematuria	6 (28.6)	21 (40.4)	0.27	14 (66.7)	36 (69.2)	0.77
Melena	2 (9.5)	4 (7.7)	1.00	6 (28.6)	10 (19.2)	0.67
Cerebral bleed	0 (0.0)	0 (0.0)	—^†^	0 (0.0)	1 (1.9)	—^†^

*Subjective or objective

^†^Cannot be calculated due to missing information.

**Fig 4:**
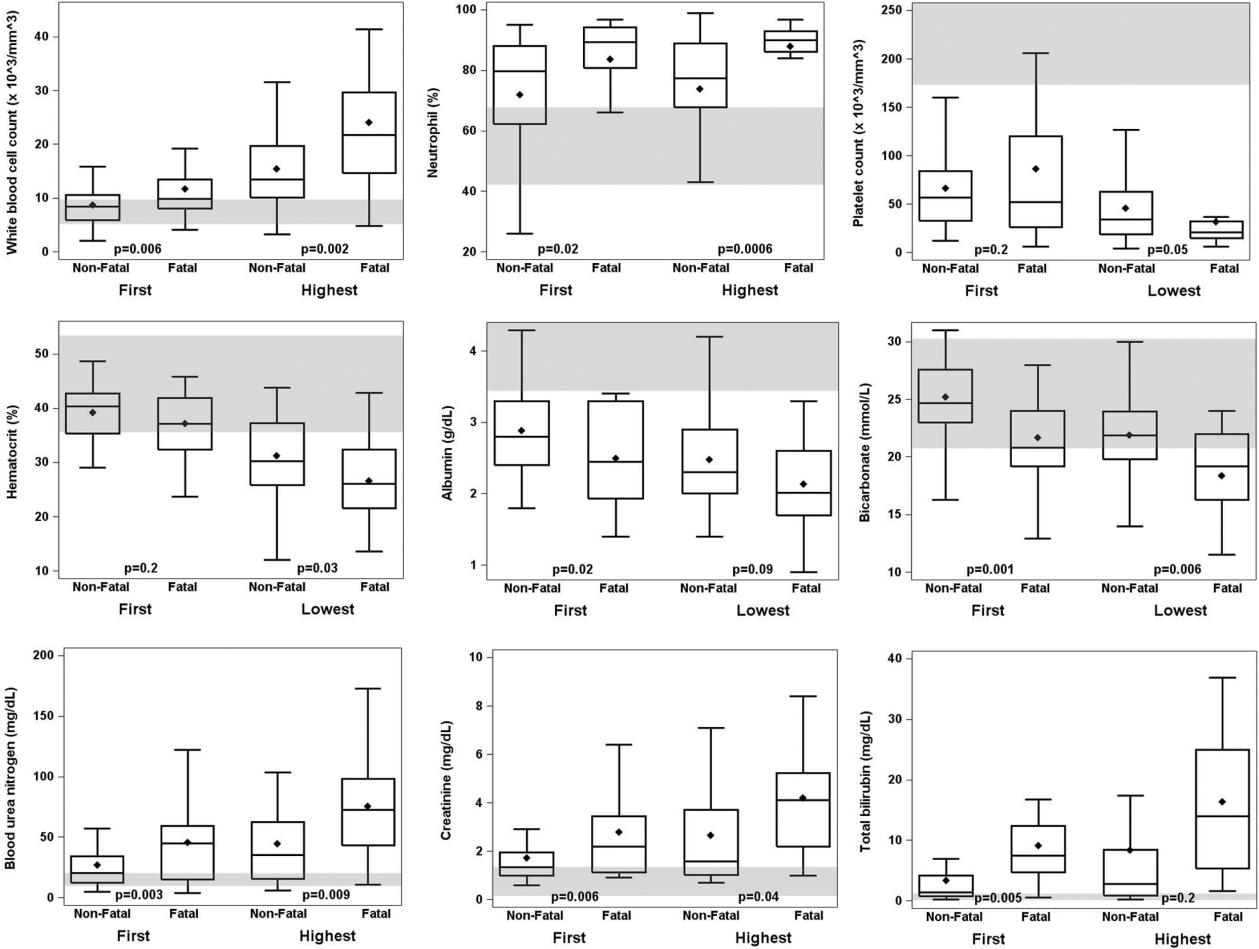
Box plots of selected laboratory values of fatal (n = 21) and non-fatal (n = 52) leptospirosis patients included in a case-control study, Puerto Rico, 2010. Medical records from all health care visits were abstracted, and first and worst laboratory values (S4 Table) were compared. The median value is indicated by the horizontal line within each box; mean value is indicated by the diamond; 25^th^ and 75^th^ interquartile range (IQR) are indicated by the bottom and top edges of the box, respectively; whiskers indicate the range of values within 1.5 times the value of the IQR. P values indicate statistical significant differences between fatal and non-fatal patients. Shaded horizontal lines indicate normal reference laboratory values.

DPO of first laboratory values did not differ significantly between cases and controls. As compared to controls, at first medical visit cases had significantly elevated white blood cell (WBC) count, proportion of neutrophils, BUN, creatinine, and total bilirubin, and decreased bicarbonate and albumin (Fig 4, S4 Table). For cases, these values were also more frequently outside of normal ranges. Throughout the clinical course, cases had significantly elevated WBC count, proportion of neutrophils, BUN, and creatinine, and decreased hematocrit, bicarbonate, albumin, prothrombin time (PT), and partial thromboplastin time (PTT).

### Multivariable model of early indicators of fatal outcome

Because fever and cough were the only early clinical signs and symptoms that were associated with fatal outcome and may be spurious findings (see Discussion), only laboratory values were included as parameters in the model. BUN and PTT were removed from the model due to higher specificity of creatinine for kidney injury as opposed to dehydration and infrequency of the test being requested at initial patient presentation, respectively. Clinical laboratory values significantly associated with fatal outcome at first presentation as compared to controls included: decreased serum bicarbonate with elevated serum creatinine, elevated WBC count, or decreased platelet count; and elevated WBC count with elevated serum creatinine (Table 3).

**Table 3:** Multivariate analysis for laboratory values associated with fatal leptospirosis patients, Puerto Rico, 2010.

Model	Clinical test 1	OR (95% CI)	P-value	Clinical test 2	OR (95% CI)	P-value	Model P-value
**1**	Decreased serum bicarbonate	0.51 (0.12–0.91)	0.006	Elevated serum creatinine	2.93 (1.03–11.72)	0.042	<0.001
**2**	Decreased serum bicarbonate	0.50 (0.21–0.81)	<0.001	Decreased platelet count	1.02 (1.00–1.05)	0.015	<0.001
**3**	Decreased serum bicarbonate	0.19 (<0.01–0.78)	<0.001	Elevated WBC count	1.25 (1.03–1.75)	0.025	<0.001
**4**	Elevated WBC count	1.17 (1.03–1.38)	0.014	Elevated serum creatinine	2.09 (1.14–4.71)	0.010	0.001

Abbreviations: OR = odds ratio; CI = confidence interval; WBC = white blood cell

## Discussion

Enhanced surveillance demonstrated a high rate of morbidity and mortality due to leptospirosis in Puerto Rico in 2010 (4.7 and 0.7 cases per 100,000 residents, respectively). Comparable incidences have been observed in other regions of the Caribbean that have conducted enhanced surveillance [32–36], which also demonstrated highest burden in older male agricultural workers and the unemployed [2, 36]. Although the patients identified in Puerto Rico reflected the expected clinical characteristics of severe leptospirosis (i.e., pulmonary hemorrhage, acute kidney injury, and/or septic shock with multi-organ failure), under recognition and underreporting of leptospirosis cases was prominent, as one-third of patients were never diagnosed with leptospirosis and two-thirds were not reported to public health authorities. These findings together demonstrate that leptospirosis remains a neglected tropical disease in Puerto Rico.

Several missed opportunities for early clinical intervention were identified in this study. First, although fatal cases sought care earlier and were more often diagnosed with leptospirosis at first medical visit; however, fatal patients less often first sought care at a hospital, and were not admitted to the hospital any sooner than non-fatal patients. Thus, delayed hospital admission may have contributed to fatal outcome, as has been previously reported [12, 13]. However, we saw no evidence that this delay was associated with the timing of initiation of antibiotic therapy, which did not differ between cases and controls. Although prospective clinical trials of antibiotics have not demonstrated a clear benefit to leptospirosis patient outcome [37], this should not preclude administration of antibiotics to patients with suspected leptospirosis [10]. Last, roughly half of all leptospirosis patients were given corticosteroids, which may result in increased risk of hemorrhage and immunosuppression. A recent systematic review demonstrated no clear benefit to leptospirosis patient outcome by administering corticosteroids [38]; however, prospective clinical trials have yet to be conducted.

To improve recognition of leptospirosis and thereby mediate earlier admission for care, clinicians should be aware of patient characteristics and clinical indicators associated with severe leptospirosis. Most previous studies that identified risk factors associated with death due to leptospirosis relied on data collected during the final medical visit, which may be suboptimal for identification of early indicators of fatal outcome. After matching for age and status of hospitalization, no patient characteristics, including gender and history of smoking [15, 39], were significantly associated with fatal outcome in this study. Similar to previously studies [5, 8, 12, 16–18], we observed that jaundice, hemoptysis, acute kidney injury, and dyspnea or respiratory insufficiency were significantly associated with fatal outcome in this study, though not at initial medical visit. Therefore, the utility of these signs and symptoms may be limited in early identification of leptospirosis patients at risk for fatal outcome. Unexpected risk factors associated with fatal leptospirosis in this study were absence of cough and fever at first health care visit and lack of development of fever throughout hospitalization. Cough at initial presentation has been previously associated with protection from fatal outcome [12], though for unclear reasons. Potential explanations for lack of fever being associated with fatal outcome include incomplete capture of fever history, self-administration of antipyretics, or earlier entry into decompensated shock. Further studies should address the association of these signs and symptoms with fatal leptospirosis.

A prominent utility of this study was the association of common clinical laboratory values with fatal leptospirosis, specifically decreased bicarbonate with decreased platelet count and increased WBC count with elevated creatinine, all of which have been previously associated with severe leptospirosis [5, 8, 16–18, 40]. However, we did not observe that elevated serum potassium either at first presentation or at any point during hospitalization was associated with fatal outcome, as has previously been reported [40–43]. Nonetheless, the values of the laboratory markers of fatal outcome identified in this study tended to be farther outside of normal ranges at first presentation in fatal as compared to non-fatal patients, suggesting that patients with fatal leptospirosis may have progressed to severe disease more rapidly. In line with this, fatal patients were more likely to be diagnosed with leptospirosis earlier than were non-fatal patients, who were more likely to ever be diagnosed with dengue. Because previous studies associated elevated WBC count and elevated serum creatinine with leptospirosis as compared to dengue [20, 44–46], these clinical laboratory values may have utility in not only differentiating leptospirosis patients from dengue patients, but also in identifying leptospirosis patients at risk for poor outcome. Future studies should evaluate the prospective benefit of using such combinations of laboratory values to improve patient outcome through early identification and admission.

Compared to previous studies that have identified risk factors associated with severe or fatal outcome in leptospirosis patients, a major strength of this study was the design of the case-control study. By reviewing medical records from each health care visits made by patients included in the case-control study, and not solely those from the patients’ hospitalization, we avoided biasing results towards points in patients’ illness in which they were likely to be more clinically severe (i.e., at point of hospitalization). This also enabled identification of clinical indicators that would be of clinical utility before patients were hospitalized, which could thereby mediate more rapid diagnosis and/or hospitalization of patients at-risk for fatal outcome. Moreover, by closely matching patients by age we avoided identification of risk factors that may be associated with older populations. These aspects of study design together may account for some differences in factors associated with fatal outcome identified by this study as compared to previous studies that did not control for age [3, 8, 12, 16, 17, 47]. Additional strengths of this study include: conducting surveillance for fatal leptospirosis cases by testing specimens collected during autopsy of patients that died following an AFI, without which many fatal cases would not have been diagnosed; and utilizing multiple surveillance systems to identify fatal and non-fatal leptospirosis patients and subsequently comparing them using a standardized instrument for chart abstraction.

Conversely, one limitation of this study is potential misclassification of some probable leptospirosis patients due to the presence of pre-existing neutralizing antibody. However, because several thousand suspected but dengue-negative cases reported to PRDH in 2010 were not tested for evidence of leptospirosis, the incidence of leptospirosis identified herein is likely an underestimate. Also, although previous studies have demonstrated that predictors of fatal leptospirosis include oliguria [8, 17, 18, 41, 48, 49] and anuria [12], we were unable to explore these factors due to the unavailability of routine clinical data on urine output. Moreover, due to limited sample size, we were also unable to identify specific cut-offs of clinical laboratory values associated with fatal outcome. Last, we were unable to evaluate DENV/*Leptospira spp*. co-infection as a risk factor for death since most leptospirosis cases were identified by screening suspected dengue cases that tested laboratory-negative for dengue.

Clinical trainings to improve early recognition of leptospirosis patients, interpretation of diagnostic test results, need for case reporting, and clinical management should be conducted among clinicians working in both out-patient and in-patient settings in Puerto Rico. Since improvements in case surveillance and clinical awareness have been associated with decreases in patient mortality due to leptospirosis [6], such trainings may also be needed in other areas of the tropics where clinical under recognition of leptospirosis may be high [2]. Population-based serosurveys should be conducted to accurately quantitate the burden of leptospirosis and identify modifiable risk factors associated with infection, including identification of the animal reservoirs that transmit *Leptospira spp*. to humans. Such findings can be used to develop educational campaigns to inform the public of population-specific strategies that can be employed to reduce their risk of leptospirosis.

## Supporting Information

S1 Checklist STROBE Checklist. (DOCX)

S1 Fig Fatal (n = 78) and non-fatal leptospirosis (n = 651) patients reported to Puerto Rico Department of Health, 1990–2014. Year of illness onset or report were plotted for leptospirosis patients reported via the Notifiable Diseases Surveillance System.
(TIF)

S1 Table Method of leptospirosis diagnosis and rule out of dengue in fatal (n = 26) and non-fatal (n = 149) leptospirosis patients, Puerto Rico, 2010. (DOCX)

S2 Table Reported causes of death* in fatal leptospirosis patients, Puerto Rico, 2010. (N = 26).
(DOCX)

S3 Table Demographics, exposure, and medical history of matched fatal and non-fatal leptospirosis patients, Puerto Rico, 2010. (DOCX)

S4 Table Laboratory values and comparison results of matched fatal and non-fatal leptospirosis patients at presentation or worst recorded value during entire hospitalization, Puerto Rico, 2010.* (DOCX)
