# Supplementary figures and images for: Early Indicators of Fatal Leptospirosis during the 2010 Epidemic in Puerto Rico
Source: PLoS Negl Trop Dis. 2016 Feb 25;10(2):e0004482. doi: 10.1371/journal.pntd.0004482 (PMC4767218; doi:10.1371/journal.pntd.0004482)

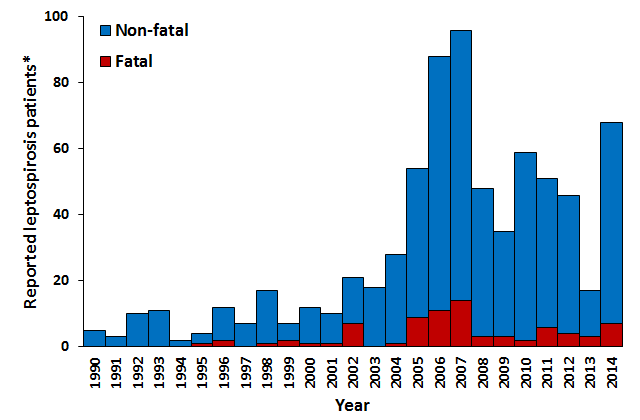

Supplement: S1 Fig — Year of illness onset or report were plotted for leptospirosis patients reported via the Notifiable Diseases Surveillance System. (TIF) [file pntd.0004482.s002.tif]
