# Supplementary material for: Early Indicators of Fatal Leptospirosis during the 2010 Epidemic in Puerto Rico
Source: PLoS Negl Trop Dis. 2016 Feb 25;10(2):e0004482. doi: 10.1371/journal.pntd.0004482 (PMC4767218; doi:10.1371/journal.pntd.0004482)
Supplement: S1 Table — (DOCX) [file pntd.0004482.s003.docx]

**S1 Table. Method of leptospirosis diagnosis and rule out of dengue in fatal (n = 26) and non-fatal (n = 149) leptospirosis patients, Puerto Rico, 2010.**

| **Method of Diagnosis** | **Fatal** | | | | **Non-fatal** | | | |
| --- | --- | --- | --- | --- | --- | --- | --- | --- |
|  | **Confirmed**  **N = 21** | | **Suspected**  **N = 5** | | **Confirmed**  **N = 91** | | **Probable**  **N = 58** | |
| **Leptospirosis**  Immunohistochemistry  PCR*  MLST  MAT  IgM ELISA†  “Leptospirosis” on death certificate | **Tested**  15  6  3  3  11  25 | **Positive**  15  3  1  2  6  15 | **Tested**  0  1  0  1  3  5 | **Positive**  --  0  --  0  0  5 | **Tested**  0  7  4  75  62  NA | **Positive**  --  4  3  70  56  -- | **Tested**  0  4  0  58  4  NA | **Positive**  --  0  --  58  0  -- |
| **Dengue**  Immunohistochemistry  RT-PCR*  IgM ELISA | 12  13  12 | 0  2  1 | 0  2  0 | --  0  -- | 0  30  51 | --  1  0 | 0  20  58 | --  0  0 |

*Blood or tissue specimen

†Three specimens positive by multiple methods

NA = not applicable; PCR = polymerase chain reaction; RT-PCR = reverse transcriptase-polymerase chain reaction; MLST = multi-locus sequence typing; IgM = immunoglobulin M antibody; MAT = microscopic agglutination test
