# Supplementary material for: Early Indicators of Fatal Leptospirosis during the 2010 Epidemic in Puerto Rico
Source: PLoS Negl Trop Dis. 2016 Feb 25;10(2):e0004482. doi: 10.1371/journal.pntd.0004482 (PMC4767218; doi:10.1371/journal.pntd.0004482)
Supplement: S2 Table — (N = 26). (DOCX) [file pntd.0004482.s004.docx]

**S2 Table. Reported causes of death* in fatal leptospirosis patients, Puerto Rico, 2010. (N = 26).**

| **Diagnosis** | **Death certificates****  **(n = 24)** | **Discharge diagnoses (n = 26)** |
| --- | --- | --- |
| Respiratory failure | 6 | 17 |
| Cardiac failure |  | 10 |
| Renal failure/acute kidney injury | 4 | 7 |
| Septic shock | 4 | 7 |
| Sepsis | 3 | 6 |
| Multi-organ failure | 4 | 6 |
| Anemia |  | 6 |
| ARDS | 4 | 4 |
| Bronchopneumonia/pneumonia/bronchitis | 1 | 4 |
| Rhabdomylysis |  | 4 |
| Pulmonary hemorrhage |  | 3 |
| Disseminated intravascular coagulopathy/Coagulopathy | 2 | 3 |
| Pulmonary edema |  | 2 |
| Seizures |  | 2 |
| Bleeding/hemorrhage | 3 | 2 |
| Metabolic acidosis |  | 2 |
| Electrolyte imbalance |  | 2 |
| Anemia | 1 | 2 |
| Hypovolemic shock |  | 2 |
| Altered mental status |  | 1 |
| Systemic inflammatory response syndrome |  | 1 |
| Hyperbilirubenemia |  | 1 |
| Cellulitis |  | 1 |
| Hepatitis |  | 1 |

*listed causes of death including leptospirosis, Weill’s disease, dengue, viral syndrome, infectious syndrome, and dehydration were omitted due to lack of specificity

**includes both Cause of Death as well as Contributing Causes of Death
