# Supplementary material for: Early Indicators of Fatal Leptospirosis during the 2010 Epidemic in Puerto Rico
Source: PLoS Negl Trop Dis. 2016 Feb 25;10(2):e0004482. doi: 10.1371/journal.pntd.0004482 (PMC4767218; doi:10.1371/journal.pntd.0004482)
Supplement: S3 Table — (DOCX) [file pntd.0004482.s005.docx]

**S3 Table.** **Demographics, exposure, and medical history of matched fatal and non-fatal leptospirosis patients, Puerto Rico, 2010.**

| **Characteristic** | **All**  **N = 73**  **n (%)** | **Fatal**  **N = 21**  **n (%)** | **Non-fatal**  **N = 52**  **n (%)** | **P value** |
| --- | --- | --- | --- | --- |
| **Sex**, male | 62 (84.9) | 18 (85.7) | 44 (84.6) | 1.000 |
| **Age in years**, median (range) | 46 (14, 85) | 44.5 (19.0, 67.0) | 48.0 (14.0, 85.0) | NA |
| **Employment** | | | | |
| Agriculture | 10 (13.7) | 4 (19.0) | 6 (11.5) | Reference |
| Housewife | 5 (6.8) | 2 (9.5) | 3 (5.8) | 1.00 |
| Other** | 26 (35.6) | 9 (42.9) | 17 (32.7) | 0.97 |
| Unknown | 6 (8.2) | 1 (4.8) | 5 (9.6) | 0.90 |
| Unemployed | 16 (21.9) | 4 (19.0) | 12 (23.1) | 0.73 |
| Disabled | 6 (8.2) | 1 (4.8) | 5 (9.6) | 0.69 |
| **Exposure history** | | | | |
| Animal | 21 (28.8) | 7 (33.3) | 14 (26.9) | 0.57 |
| Rat | 11 (15.1) | 5 (23.8) | 6 (11.5) | 0.29 |
| Dog | 8 (11.0) | 3 (14.3) | 5 (9.6) | 0.42 |
| Mice | 5 (6.8) | 1 (4.8) | 4 (7.7) | 1.00 |
| Other* | 6 (8.2) | 2 (9.5) | 4 (7.7) | 0.63 |
| Water | 2 (2.7) | 0 (0.0) | 2 (3.8) | - |
| Soil | 5 (6.8) | 0 (0.0) | 5 (9.6) | - |
| **Medical history** | | | | |
| Smoking | 26 (35.6) | 7 (33.3) | 19 (36.5) | 1.00 |
| Diabetes | 14 (19.2) | 3 (14.3) | 11 (21.2) | 0.48 |
| Obesity | 21 (28.8) | 5 (23.8) | 16 (30.8) | 0.56 |
| Hypertension | 22 (30.1) | 7 (33.3) | 15 (28.8) | 1.00 |
| Lung disease | 11 ()15.1 | 2 (9.5) | 9 (17.3) | 0.30 |
| Asthma | 8 (11.0) | 2 (9.5) | 6 (11.5) | 1.00 |
| Chronic obstructive pulmonary disease | 4 (5.5) | 0 (0.0) | 4 (7.7) | 0.30 |
| Unspecified | 1 (1.4) | 0 (0.0) | 1 (1.9) | 1.00 |
| Liver disease | 7 (9.6) | 2 (9.5) | 5 (9.6) | 0.65 |
| Liver cirrhosis | 2 (2.7) | 1 (4.8) | 1 (1.9) | 1.00 |
| Hepatitis B virus infection | 1 (1.4) | 0 (0.0) | 1 (1.9) | 1.00 |
| Hepatitis C virus infection | 6 (8.2) | 2 (9.5) | 4 (7.7) | 1.00 |
| Cardiac disease | 10 (13.7) | 2 (9.5) | 8 (15.4) | 0.45 |
| Co-infection | 13 (17.8) | 5 (23.8) | 8 (15.4) | 0.48 |
| Liver pathogen | 7 (9.6) | 2 (9.5) | 5 (9.6) | 1.00 |
| Other pathogen*** | 3 (4.1) | 1 (4.8) | 2 (3.8) | 1.00 |
| Respiratory pathogen | 5 (6.8) | 3 (14.3) | 2 (3.8) | 0.14 |

* = chickens, horses, pigs

** = Carpenter, construction, police/security, incarcerated

*** = dengue virus, Group G beta hemolytic *Streptococcus*, *Candida albicans*; nosocomial infections were excluded
