# Supplementary material for: Early Indicators of Fatal Leptospirosis during the 2010 Epidemic in Puerto Rico
Source: PLoS Negl Trop Dis. 2016 Feb 25;10(2):e0004482. doi: 10.1371/journal.pntd.0004482 (PMC4767218; doi:10.1371/journal.pntd.0004482)
Supplement: S4 Table — (DOCX) [file pntd.0004482.s006.docx]

**S4 Table. Laboratory values and comparison results of matched fatal and non-fatal leptospirosis patients at presentation or worst recorded value during entire hospitalization, Puerto Rico, 2010.***

| **Variable** | **At first presentation** | | | | | |  | **Worst value†** | | | |
| --- | --- | --- | --- | --- | --- | --- | --- | --- | --- | --- | --- |
|  |  | **Fatal** | **Non-fatal** | |  |  |  | **Fatal** | **Non-fatal** |  |  |
|  | **n** | **Median (Range)** | **n** | **Median  (Range)** | **OR**  **(95% CI)** | **P-value** |  | **Median  (Range)** | **Median  (Range)** | **OR**  **(95% CI)** | **P-value** |
| White blood cell (WBC)count (x 10^3^) | 21 | 9.9  (4.1–26.9) | 52 | 8.4  (2.1–23.4) | 1.18  (1.04–1.37) | 0.006 |  | 21.7  (4.8–65.3) | 13.4  (3.3–48.8) | 1.1 (1.03–1.19) | 0.002 |
| Neutrophil (%) | 21 | 89.3  (5.9–96.8) | 52 | 79.7  (26.0–95.1) | 1.04  (1.005–1.08) | 0.02 |  | 90.0  (66.0–96.8) | 77.4  (16.0–99.0) | 1.08 (1.03–1.15) | 0.0006 |
| Platelet count (x 10^3^) | 21 | 52  (6–343) | 52 | 56.5  (12–202) | 1.01  (0.996–1.02) | 0.2 |  | 21  (6–98) | 34  (4–202) | 0.98 (0.96–1) | 0.05 |
| Hematocrit (%) | 21 | 37.1  (23.7–45.8) | 52 | 40.4  (20.9–54.6) | 0.94  (0.85–1.04) | 0.2 |  | 26.1  (13.6–42.8) | 30.2 (12.0–43.8) | 0.92 (0.84–0.99) | 0.03 |
| Potassium | 21 | 3.9  (3.0–7.0) | 52 | 3.8  (2.8–6.0) | 1.27  (0.67–2.39) | 0.4 |  | 3.3  (2.6–4.3) | 3.2  (2.4–5.6) | 1.26 (0.51–3.14) | 0.6 |
| Bicarbonate | 17 | 20.8  (12.9–28.0) | 52 | 24.7  (16.3–31.0) | 0.64  (0.44–0.83) | <.0001 |  | 19.2  (11.5–24.0) | 21.9  (11.0–30.4) | 0.79 (0.62–0.95) | 0.006 |
| Calcium | 18 | 8.1  (6.9–9.4) | 52 | 8.4  (6.6–9.8) | 0.52  (0.23–1.12) | 0.1 |  | 7.4  (6.2–8.9) | 7.4  (4.3–9.1) | 1.09 (0.63–1.98) | 0.8 |
| Blood urea nitrogen (BUN) | 21 | 45.0  (3.7–168.0) | 52 | 20.5  (5.0–78.5) | 1.05  (1.01–1.09) | 0.003 |  | 72.5  (11.0–173.0) | 35.5  (6–242) | 1.02 (1.004–1.04) | 0.009 |
| Creatinine | 20 | 2.2  (0.9–10.3) | 52 | 1.3  (0.6–6.0) | 2.09  (1.18–4.32) | 0.006 |  | 4.1  (1.0–10.3) | 1.6  (0.7–13.4) | 1.26 (1.01–1.64) | 0.04 |
| Albumin | 14 | 2.4  (1.4–3.4) | 47 | 2.8  (1.8–4.3) | 0.3  (0.08–0.86) | 0.02 |  | 2.0  (0.9–3.3) | 2.3  (1.4–4.2) | 0.43 (0.14–1.13) | 0.09 |
| Alanine transaminase (ALT) | 17 | 111  (29–823) | 48 | 90  (15–5,131) | 1.0  (0.998–1.003) | 0.5 |  | 285  (56–823) | 125  (23–10,162) | 1.0 (<1–1.0005) | 0.6 |
| Aspartate transaminase (AST) | 16 | 64  (27–561) | 46 | 77.5  (16–5614) | 1.0  (0.99–1.004) | 0.9 |  | 102  (36–1,350) | 114  (16–5,614) | 1.0 (<1–1.001) | 0.7 |
| Total bilirubin | 17 | 7.5  (0.5–36.9) | 46 | 1.4  (0.2–26.3) | 1.17  (1.04–1.37) | 0.005 |  | 14.0  (1.6–36.9) | 2.8  (0.2–83.0) | 1.03 (0.99–1.08) | 0.2 |
| Prothrombin time (PT) | 17 | 12.5  (10.7–26.0) | 44 | 13.1  (10.4–18.5) | 1.15  (0.89–1.52) | 0.3 |  | 13.2  (1.3–45.9) | 13.2  (10.4–23.5) | 1.15 (1.01–1.38) | 0.03 |
| Partial thromboplastin time (PTT) | 16 | 31.2  (22.4–92.1) | 44 | 29.7  (20.0–39.4) | 1.08  (1.00–1.22) | 0.05 |  | 32.2  (14.1–180.0) | 30.3  (20.0–40.2) | 1.06 (1.01–1.2) | 0.005 |
| International normalized ratio (INR) | 16 | 1.1  (0.9–2.0) | 43 | 1.1  (0.9–1.7) | 1.4  (0.04–40.93) | 0.8 |  | 1.2  (1.0–50.7) | 1.1  (1.0–2.1) | 4.18 (0.92–40.7) | 0.07 |
| Urine specific gravity | 18 | 1.01  (1.00–1.03) | 43 | 1.02  (1.01–1.03) | <0.001  (<0.001–>1000) | 0.1 |  | 1.01  (1.00–1.03) | 1.02 (1.01–1.03) | >1000 (<1–>1000) | 0.7 |
| Urine pH | 20 | 6.0  (5.0–7.0) | 49 | 5.5  (5.0–7.5) | 1.26  (0.56–2.76) | 0.6 |  | 5.3  (5.0–8.0) | 5.5  (5.0–7.5) | 1.13 (0.57–2.16) | 0.8 |

* Only cases and controls with test results from more than one day were included.

†For WBC count, neutrophil, BUN, creatinine, ALT, AST, total bilirubin, PT, PTT, INR, and urine specific gravity, “worst” was defined by the highest value; for platelet count, hematocrit, potassium, bicarbonate, calcium, albumin, and urine pH, “worst” was defined by the lowest value.
